# Role of Galectin-1 in the Pathogenesis of Melanoma

**DOI:** 10.26502/jcsct.5079279

**Published:** 2025-12-26

**Authors:** Grace Kim, Thea Blackwell, Kimiya Banai, Rowen Lin, Justin Chung, Devendra K. Agrawal

**Affiliations:** Department of Translational Research, College of Osteopathic Medicine of the Pacific, Western University of Health Sciences, Pomona, California 91766 USA

**Keywords:** Angiogenesis, Cancer, Carbohydrate recognition domains, Endothelial cells, Fibroblasts, Galectin-1, Glycans, Immune cells, Immune invasion, Immunosuppression, Melanoma, Metastatic melanoma, Stromal cells, Tumor-associated macrophages, Tumor microenvironment

## Abstract

Galectins (Gal) are β-galactoside–binding endogenous lectins involved in a wide variety of angiogenic and immune functions throughout many tissues. Galectin 1 (Gal-1) is an important member of the galectin family, acting as a central regulator in immune cells, tumor immune evasion, metastasis, angiogenesis, and therapy resistance, especially in melanoma. Within the immune system, galectin-1 influences the activation of dendritic cells, natural killer cells, B-cells, and T-cells and their downstream effects, as well as influences myeloid-derived suppressor cells and tumor-associated macrophages. Further, Gal-1 influences cancer biology by promoting and regulating angiogenesis through the VEGFR2/NRP1 glycan binding signaling pathway and regulation of alternative splicing, and metastasis, priming pre-metastatic niches. Within melanoma, Gal-1 has a key role in therapy resistance, resisting MAPK inhibitors and chemotherapy, and cancer progression, including promoting immune evasion, SOX10-linked plasticity, and VEGF-independent angiogenesis. Therapeutic strategies have been developed to target the role of Galectin-1, including small-molecule and mAb Gal-1 inhibitors and positron emission tomography imaging for noninvasive profiling of tumor microenvironment. Despite its therapeutic potential, its vast role within different organ systems, including providing cardioprotective effects, immune system regulation, stress regulation, and axonal growth and regeneration, limits Gal-1 targeted therapies. Thus, further research is warranted to determine strategies to isolate therapeutic agents within the cancer cells. As Galectin-1 plays a role in the prognosis and treatment of melanoma, we critically reviewed the published information on its role within immune, endothelial, and stromal cells, the influence of Gal-1 on cancer biology, specifically within melanoma, therapeutic strategies to target Gal-1, and identified key gaps in current therapies and research.

## Introduction

Galectins (Gal) are a subtype of endogenous lectins involved in a wide variety of functions throughout various tissues in the body and many galectins have been implicated in cancer progression and prognosis. Through one or two conserved carbohydrate recognition domains (CRDs), galectins bind glycans [1]. Within cells, they are involved in many processes, including cell growth, signal transduction, and apoptosis [2–4]. While most galectins are found within human tissue, Galectin-5, Galectin-6, Galectin-11, and Galectin-15 are not found within human tissues. There are three classes of galectins: prototypical, tandem-repeats type, and chimera. The prototypical galectins contain one carbohydrate recognition domain and can present as homodimers. Galectins that belong within this class include Galectin-1, -2, -5, -7, -10, -11, and -13 through -16. Gal-2 is expressed in the gastrointestinal tract and placenta [5]. Gal-7 is found within stratified epithelia, skin, fetal heart, and gastrointestinal tract [6]. Gal-10 is expressed within eosinophils [7]. Gal-13 and Gal-16 are expressed within placenta and Gal-14 is expressed within eosinophils and placenta [8, 9]. The tandem-repeat type contains two carbohydrate recognition domains, joined by a functional linker peptide, and includes galectin-4, -6, -8, -9, and -12. Gal-4 can be found in the gastrointestinal tract and hippocampal and cortical neurons. Gal-8 is found within many tissues, including the brain, liver, kidney, cardiovascular system, lung, and spleen. Gal-9 is found within skin, gastrointestinal system, liver and thymus and Gal-12 is found in adipose tissue [10]. Lastly, Gal-3 belongs within the chimera type class of galectins, containing an N-terminal non-lectin region joined with a carbohydrate recognition domain [2, 11]. As many of these galectins have roles in immunomodulation and inflammation, several of them have been implicated in various cancers. For instance, in pancreatic adenocarcinoma, Galectin-9 demonstrates increased expression proportional to the stage of precancerous lesions in mice [12]. As galectins have a role in tumorigenesis, targeted immune checkpoint inhibition has shown promise in limiting progression of tumor and improving prognosis. Overall, galectins play key roles within various tissues (Table 1).

### The Role of Galectin-1 In Various Cell Types

#### Immune Cells

In the immune system, Gal-1 exerts notable immunosuppressive and regulatory effects across various immune cell types, including B cells, T cells, natural killer cells, macrophages, and dendritic cells. To produce its effect on these various cells, Gal-1 must first present a carbohydrate recognition domain (CRD) to β-galactosides, including N-acetyllactosamine (LacNAc). This Gal-1-glycan interaction is required to exert downstream effects on various cell types, such as immune cells [65]. Gal-1 plays a role in regulating B cells and, thus, inflammatory responses. Specifically, Gal-1 has a role in B cell development and differentiation, influencing different B cell subsets in different manners [66]. Galectin-1 induces regulatory B cell and IL-10 expression and suppresses TNF-α production [66].

Within dendritic cells (DCs), Gal-1 binds CD43 and CD45 receptors on their surface, inducing several regulatory effects on DC function and promoting DC migration through the extracellular matrix [67]. The effects of Gal-1 on DCs include DC phenotype modulation, enhancing their tolerogenic properties, and triggering complex immunomodulatory effects on DCs [68]. Džopalić et al. [69] demonstrated that Gal-1 modulates the phenotype, stimulating their maturation, and function of human monocyte-derived dendritic cells. Gal-1 promotes a tolerogenic profile by downregulating co-stimulatory molecule expression and reducing pro-inflammatory cytokine production. Similarly, Gal-1 has been shown to suppress osteoclastic differentiation, suggesting a role in immune cell-driven bone remodeling. Specifically, Takeuchi et al. [70] found that the reduced form of Gal-1 inhibited osteoclastogenesis in both human peripheral blood mononuclear cells and murine RAW264 cells.

In natural killer (NK) cells, Gal-1 indirectly regulates cytotoxic function through the miR1983-TLR7-IFNβ signaling axis. This pathway, identified by Shah et al. [71], facilitates NK cell-mediated glioma cell killing and is modulated by Gal-1 expression.

Gal-1 produces regulatory effects on the differentiation, cytokine production, and survival of several T cell subpopulations. Gal-1 is highly expressed in regulatory T cells, especially after activation, and is associated with the production of Treg cells [72]. Gal-1 positively regulates CD4+ T cell maturation into Th2 and Treg cells, as demonstrated by Yakushine et al. [72] Within Th17 and Th1 cells in contrast, Gal-1 promotes apoptosis [73]. Further, De la Fuente et al. [74] demonstrate that Gal-1 is a key regulator in Th17 differentiation and function. In regards to its regulation of cytokine production, Gal-1 is associated with the production and suppression of several cytokines. For instance, Gal-1 exerts inhibitory effects on T cell production of IL-2 [75], IFN-γ [73, 75], and IL-17. However, within Th2 and Treg cell populations, the presence of Gal-1 is also positively associated with anti-inflammatory cytokine production[73]. Furthermore, Gal-1 expression in CD8+ T cells plays a crucial role in controlling inflammatory responses in contact hypersensitivity, supporting the broader role of Gal-1 in immune homeostasis and inflammation resolution [16]. The regulatory effects of Gal-1 are modulated by the glycosylation status of various glycoprotein receptors on T cells, including CD43 and CD45. For example, N- and O-glycan status of CD45 and CD43 receptors modifies the ab ility of Gal-1 to induce apoptosis in T cells [76].

#### Endothelial Cells

In endothelial cells, Gal-1 exhibits dual roles depending on the context and cellular environment. High concentrations of Gal-1 and Gal-3 significantly inhibit angiogenic activity in human retinal microvascular endothelial cells in vitro, indicating anti-angiogenic potential [77]. Conversely, in a study of pyogenic granulomas, Arciniegas et al. observed Gal-1 expression in plump endothelial cells contributing to the formation of new vascular structures, suggesting a pro-angiogenic or remodeling function under certain pathological conditions. This duality highlights the context-dependent influence of Gal-1 on endothelial function and vessel formation.

#### Fibroblasts and Stromal Cells

**Figure 1:**
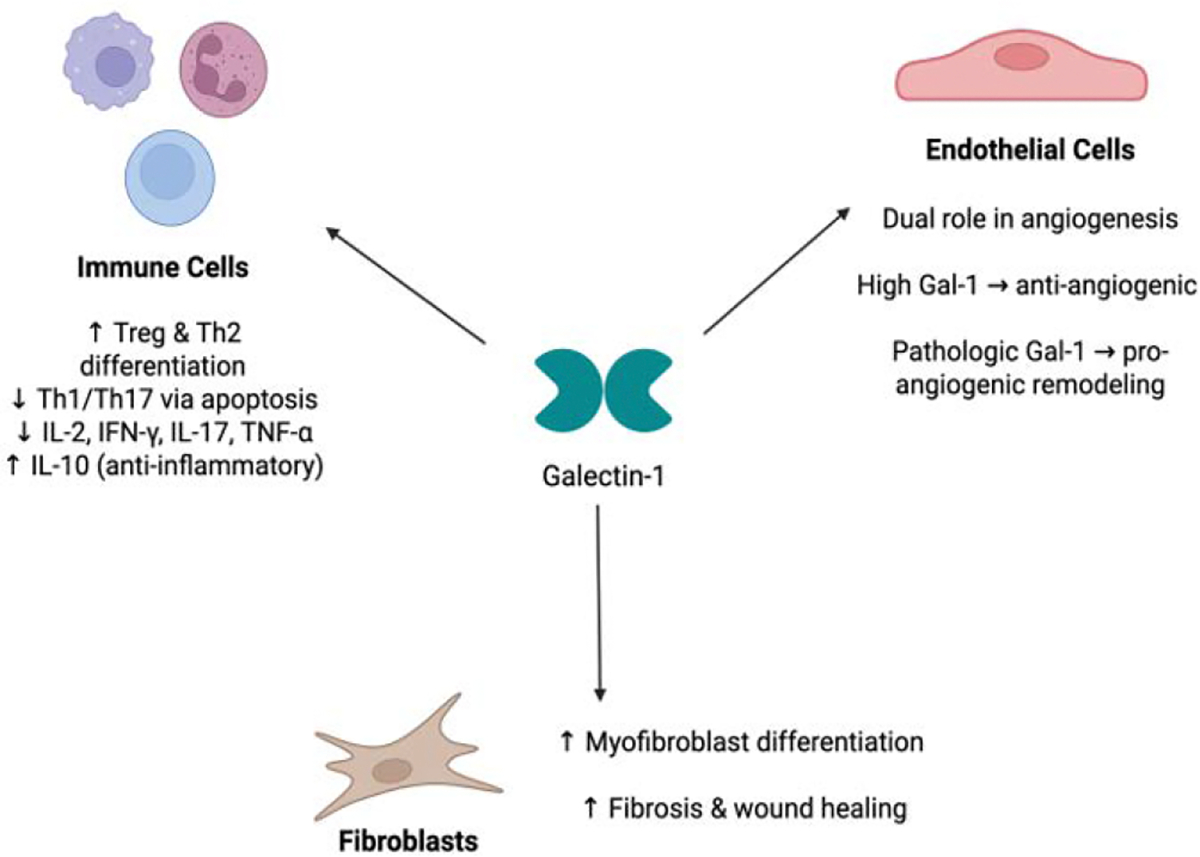
Role of Gal-1 in Different Immune and Structural Cell Types. Galectin-1 (Gal-1) exerts diverse regulatory effects in multiple cell populations. In immune cells, Gal-1 promotes Treg and Th2 differentiation while suppressing Th1/Th17 subsets and pro-inflammatory cytokines, thereby enhancing anti-inflammatory signaling. In endothelial cells, Gal-1 demonstrates a dual role in angiogenesis—high Gal-1 levels inhibit angiogenic activity, whereas pathologic Gal-1 expression promotes neovascular remodeling. In fibroblasts, Gal-1 induces myofibroblast differentiation and enhances fibrosis and wound-healing responses.

In fibroblasts and stromal cells, Gal-1 is closely linked to fibrosis and wound healing. The elevated expression of Gal-1 in hypertrophic scar tissue, as shown by Kirkpatrick et al., suggests its involvement in fibrotic skin remodeling. Similarly, pharmacological inhibition of Gal-1 reduced lung fibroblast activation and proliferation, indicating its contributory role in pulmonary fibrosis [78]. Weng et al. [79] further elucidated a mechanistic pathway through which Gal-1 promotes fibroblast-to-myofibroblast differentiation via cytochrome P450 activation in pulmonary fibrosis models. In addition to its role in fibrotic processes, Gal-1 modulates insulin-like growth factor-1 activity in neonatal skin fibroblasts in the context of galactosemia, linking Gal-1 to fibroblast-mediated metabolic and developmental processes [80]. Intracellularly, Gal-1 directly binds to the proteinaceous core of fibroblast growth factor 12 (FGF12) and acts as a down-regulator to its activity [81]. FGF12 is also found in neurons, osteoclasts, and cardiomyocytes, and its dysregulation has been implicated in diseases of the nervous system, cancers, and cardiac diseases [82] Thus, the role of Gal-1 in intracellular and extracellular signaling of fibroblasts and stromal cells implicates it in many fibrotic and proliferative diseases (Figure 1).

### Role of Galectin-1 in Cancers

#### Immune Evasion and Immunosuppression

Galectin-1 (Gal-1) plays a multifaceted role in cancer progression, prominently through immune evasion, angiogenesis, metastasis promotion, and resistance to therapy. Elevated Gal-1 expression fosters immune suppression within the tumor microenvironment (TME) by increasing populations of regulatory T cells (Tregs) and other immunosuppressive cells [83, 84]. Specifically, Gal-1 enhances the infiltration and immunosuppressive function of CD8+CD122+PD-1+ Tregs and myeloid-derived suppressor cells (MDSCs), thereby diminishing effector T cell responses [85, 86]. Gal-1 also binds to glycosylated receptors such as LAG-3, TIM-3, and PD-1 on T cells, contributing to T cell exhaustion and reduced cytotoxic activities [86]. Recent findings further underscore the immunomodulatory influence of Gal-1, demonstrating that Gal-1 promotes a tumor-associated macrophage (TAM) phenotype by upregulating key immunosuppressive markers such as programmed cell death ligand 1 (PD-L1) and indoleamine 2,3-dioxygenase-1 (IDO1) via the JAK/STAT signaling pathway [87]. Gal-1-treated macrophages also increased production of the immunosuppressive metabolite kynurenine, reinforcing its role in shaping a suppressive TME [87]. Notably, Gal-1 was found to be enriched in the tumor extracellular matrix and derived from both epithelial and stromal cells, emphasizing its pervasive influence across tumor compartments. Reduction of Gal-1 levels, as mediated by KLF12, has been shown to boost CD8+ T cell infiltration and function within tumors, highlighting its immunomodulatory potential [88]. Furthermore, targeting Gal-1 with specific inhibitors like GB1908 could potentiate cytotoxic T cell responses, suggesting its therapeutic utility in cancer immunotherapy [89].

#### Angiogenesis and Metastasis Promotion

Additionally, Gal-1 contributes to angiogenesis and metastasis. Research has shown that internalization of Gal-1 via microvesicles substantially promotes cancer cell migration, emphasizing the importance of stromal-derived Gal-1 in tumor motility [90]. Interaction between Gal-1 and glucose-regulated protein 78 (GRP78) notably enhances proliferation and metastatic potential in gastric cancer cells [91]. Moreover, the critical role of Gal-1 in angiogenesis is highlighted by studies demonstrating reduced endothelial cell proliferation, migration, and tube formation when Gal-1 is knocked out in antlerogenic periosteal cells [92]. Gal-1 fosters pre-metastatic niche formation by accumulating polymorphonuclear myeloid-derived suppressor cells (PMN-MDSCs) in distant organs such as the lungs, facilitating metastatic spread [93]. Gal-1 binds to glycans on the surfaces of VEGFR2 and neuropilin, activating pro-angiogenic signaling pathways. Further, VEGF levels have been correlated with cancer staging and possibly prognosis. Throughout these mechanism, Gal-1 promotes angiogenesis [94]. Regarding its role in angiogenesis, Galectin-1 triggers signaling of vascular and anti-angiogenic responses through regulation of genes at transcriptional and post-transcriptional levels [95]. Gal-1 interacts with the mRNAs of VEGFA, EGR1, and LAMA5, which are genes that play vital roles in angiogenic responses. Gal-1 also inhibits capillary tube formation in an in vitro study, indicating its role within regulation of angiogenesis [96].

#### Tumor Progression and Resistance to Therapy

Gal-1 also influences tumor progression and resistance to therapeutic interventions. ZFP64 has been shown to transcriptionally upregulate Gal-1, promoting stem cell-like features and contributing to an immunosuppressive gastric cancer microenvironment Moreover, TREM2+ tumor-associated macrophages (TAMs) secrete Gal-1, leading to PD-L1 overexpression in vascular endothelial cells and subsequent impairment of CD8+ T cell infiltration [97]. Clinically, elevated Gal-1 is linked to reduced responsiveness to immunotherapies, characterized by higher infiltration of Tregs and MDSCs and decreased CD8+ T cell infiltration [98]. Gal-1 further mediates resistance to chemotherapy; notably, it enhances cisplatin resistance in gastric carcinoma via the NRP-1/C-JUN/Wee1 signaling pathway, an effect that can be reversed by the inhibitor EG00229 [99]. Additionally, fibroblast-derived Gal-1 significantly contributes to drug resistance and the maintenance of cancer-initiating cells in colorectal cancer [100]. The association between elevated Gal-1 and Gal-3 expression with aggressive tumor behaviors, metastasis, and poorer prognosis has been particularly noted in hepatocellular carcinoma, emphasizing the role of Gal-1 as a prognostic indicator and therapeutic target in cancer management [101]. Additionally, Gal-1 also effect CD45+ cells. Within normal tissue, CD45+ cells promote ECM formation by secreting matrix metalloproteinases. However, Gal-1 expression alters CD45+ role in tissue remodeling at the margins of tumors, promoting tumor growth [102].

### Melanoma: Pathogenesis and Role of Galectin-1

#### Galectin-1 and Melanoma Progression

Melanoma pathogenesis involves interactions where Gal-1significantly influences progression, angiogenesis, and therapy resistance. Gal-1 contributes significantly to melanoma progression by suppressing Th1 and Th17 immune responses, thereby facilitating immune evasion [103]. Moreover, Gal-1 upregulates SOX10, a transcription factor critical in neural crest cell induction, further enabling melanoma cells to evade immune detection [104]. The diagnostic significance of SOX10 is further underscored by findings in triple-negative breast carcinoma (TNBC), where SOX10 is expressed in 60% of cases, particularly those with poorly differentiated, aggressive features, highlighting its association with neural crest-like and stem-like phenotype [105, 106]. In melanoma, such expression patterns likely reflect Gal-1–driven transcriptional programming that supports tumor plasticity and immune escape. The tumor microenvironment (TME) is influenced by melanoma-derived exosomal miR-125b-5p, which targets Lysosomal Acid Lipase A (LIPA), altering tumor-associated macrophages (TAMs) toward a pro-tumor phenotype [107]. miR-125b-5p has also been implicated in promoting cancer aggressiveness by modulating the Sema4D signaling axis, as demonstrated in colorectal cancer, where the suppression of miR-125b-5p on Sema4D enhanced cell invasion and proliferation [108]. Given the role of Sema4D in immune modulation and tumor-stroma interactions, these findings suggest that exosomal miR-125b-5p may promote melanoma progression not only by reprogramming macrophages but also by silencing anti-tumor pathways like Sema4D. Recent findings reveal that the TRAF3/STAT6 signaling axis is a key regulator of macrophage polarization, where loss of TRAF3 enhances STAT6 activation, driving M2 polarization and promoting tumor progression [106]. These mechanisms likely synergize with Gal-1–driven signals in the TME, contributing to macrophage-mediated immune suppression and tumor growth. Additionally, transcriptomic analysis indicates that TAMs specifically upregulate Gal-1, suppressing T cell function and promoting immune escape [109]. Furthermore, Galectin-3 (Gal-3) modulates Notch signaling, promoting melanoma growth and metastatic potential.

#### Galectin-1 and Angiogenesis in Melanoma

**Figure 2:**
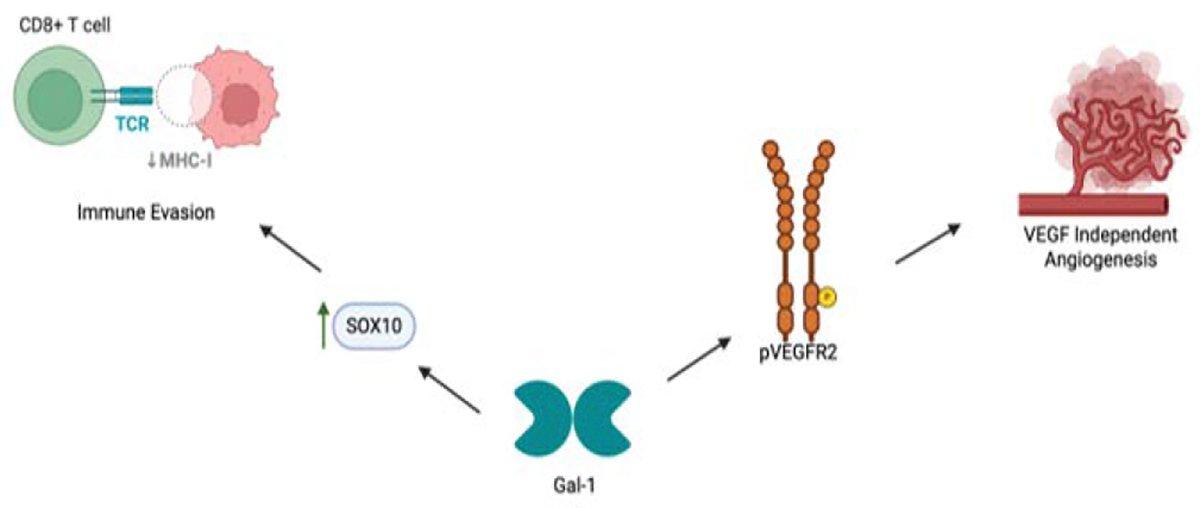
Role of Gal-1 in Melanoma Pathogenesis. Schematic representation of Galectin-1 (Gal-1)–mediated mechanisms promoting immune evasion and VEGF-independent angiogenesis in melanoma. Upregulation of SOX10 enhances Gal-1 expression, which contributes to tumor immune escape by downregulating MHC-I expression on tumor cells, reducing CD8^+^ T cell recognition. Concurrently, Gal-1 activates phosphorylated VEGFR2 (pVEGFR2), driving angiogenesis independent of VEGF signaling.

Gal-1 plays a significant role in melanoma-associated angiogenesis. It enhances endothelial cell migration, tubulogenesis, and VEGFR2 phosphorylation, critical steps for tumor angiogenesis [94]. Gal-1 further enables melanoma resistance to anti-VEGF therapies like bevacizumab by sustaining angiogenic processes independent of VEGF signaling [94, 110]. Similar VEGF-independent angiogenic mechanisms have been identified in pancreatic cancer, where BICC1 was shown to drive tumor progression by upregulating angiogenesis-related genes and activating ERK signaling, thereby promoting angiogenesis in a VEGF-independent manner [111]. Similarly, in the context of retinal neovascularization, a Gal-1-dependent competing endogenous RNA (ceRNA) network in human retinal microvascular endothelial cells (HRMECs), demonstrating the regulatory role of Gal-1 over angiogenesis-related gene expression at the post-transcriptional level [112]. Collectively, these findings indicate that Gal-1 not only facilitates angiogenesis across diverse tissues and disease contexts but also functions through multifaceted and VEGF-independent mechanisms (Figure 2).

Clinically, resistance to anti-angiogenic therapies remains a substantial challenge in melanoma. In metastatic mucosal melanoma, standard chemotherapy, biochemotherapy, and anti-VEGF therapies have demonstrated limited efficacy, often due to compensatory activation of alternative pro-angiogenic pathways like those mediated by Gal-1 [113]. Nevertheless, VEGF-targeted strategies may still provide therapeutic benefits when used in combination with immunotherapy. For instance, VEGF blockade amplifies tumor hypoxia, which paradoxically enhances CD8^+^ T cell infiltration and antitumor activity [114].

Building on this, combining VEGF-pathway inhibition with anti–PD-1 therapy can further augment antitumor immunity in melanoma [115]. Notably, the combination of lenvatinib—a multi-kinase inhibitor targeting VEGFR1–3, FGFR1–4, and others—with anti–PD-1 therapy was more effective than anti-VEGF antibody plus anti–PD-1. Lenvatinib promoted greater immune activation, enhanced T cell infiltration, and superior tumor regression, suggesting that the choice of VEGF-pathway inhibitor critically shapes the immunologic and therapeutic response [115].

A combination therapeutic approach targeting VEGF, alongside SOX10 silencing, significantly improves antiangiogenic effects, suggesting potential for enhanced therapeutic efficacy in melanoma models both in vitro and in vivo [116].

#### Galectin-1 and Resistance to Melanoma Therapy

In the context of therapeutic resistance, Gal-1 is increasingly recognized for its critical contribution. Gal-1 facilitates tumor-associated neovascularization, thereby contributing to melanoma aggressiveness and therapeutic resistance [110]. Vaccination against Gal-1 enhances cytotoxic T-cell infiltration and notably reduces tumor burden, highlighting its potential as an immunotherapeutic target [117]. Notably, BRAF inhibitor treatments, while decreasing PD-L1 expression, paradoxically increase Gal-1 levels, thus fostering an immunosuppressive TME [118]. Gal-1 upregulation has been observed in melanoma cells resistant to BRAF-targeted therapies, significantly contributing to therapeutic resistance [119]. Mechanistically, Gal-1 facilitates melanoma progression through Akt phosphorylation, which activates the mTOR pathway, thereby enhancing cellular metabolism, proliferation, and promoting epithelial-mesenchymal transition (EMT [120]. The support of Gal-1 for sustained Akt signaling enables melanoma cells to circumvent MAPK inhibition and maintain their proliferative capacity, thereby reducing the effectiveness of immunotherapy Complementary studies identify TRIM14 as a promoter of melanoma malignancy through the PTEN/PI3K/Akt pathway, further emphasizing the critical role of the PI3K/Akt pathway in therapeutic resistance.

Recent research has highlighted the multiple, intersecting mechanisms that underlie acquired resistance. CoREST repressor complex as a key mediator of phenotype switching, enabling melanoma cells to toggle between drug-sensitive and drug-resistant states [121]. This epigenetic plasticity facilitates immune evasion and resistance to MAPK inhibitors. Disruption of CoREST impaired phenotype switching, restoring therapy responsiveness and T-cell-mediated cytotoxicity.

Similarly, acquired resistance reprograms the TME by impairing antigen presentation and enriching immunosuppressive myeloid cells, contributing to cross-resistance to immune checkpoint blockade [122]. In alignment with these findings, CCR2^+^ monocyte-derived macrophages dynamically shape the trajectory of resistance to BRAF-targeted therapy [123]. These macrophages infiltrate tumors during therapy and secrete factors that promote survival of drug-tolerant melanoma cells, ultimately supporting the emergence of fully resistant clones. Depletion or blockade of CCR2^+^ macrophages disrupted this process, delayed resistance, and enhanced therapeutic efficacy, emphasizing the critical role of macrophage-driven TME remodeling in resistance evolution.

Furthermore, genomic instability, mediated by Aurora kinase A (AURKA), is a distinct driver of acquired resistance through chromosomal missegregation and DNA damage [124]. Targeting AURKA stabilized the genome and significantly delayed the development of resistance in preclinical models.

Together, these findings support a comprehensive model of resistance in melanoma, wherein Gal-1-driven immune suppression, PI3K/Akt/mTOR signaling, CoREST-mediated phenotype switching, CCR2^+^ macrophage-dependent microenvironmental cues, and AURKA-induced genomic instability act in concert to promote therapeutic escape. Rational, multi-pronged strategies targeting these interconnected mechanisms may be essential to preventing or overcoming resistance to both targeted and immune therapies.

### Therapeutic Strategies of Inhibiting Galectin-1

Targeting Gal-1 has emerged as a promising strategy to enhance anti-tumor immune responses and improve outcomes in melanoma treatment. Gal-1 plays a crucial role in immune evasion by suppressing T-cell activity and fostering an immunosuppressive tumor microenvironment, making it an attractive target for melanoma immunotherapy [117]. Recent advancements in molecular imaging, such as Galectin-1 PET imaging, have enabled noninvasive monitoring of Gal-1 expression in melanoma tumors, providing valuable insights into the tumor microenvironment and helping predict immunotherapy responses [125].

However, a significant challenge in melanoma treatment arises with the use of BRAF inhibitors, which can lead to an upregulation of Gal-1, fostering resistance and reducing therapeutic efficacy. This necessitates the development of combination strategies to counteract this compensatory mechanism [118]. Research in other cancers, such as thyroid cancer, has shown that targeting Gal-1 can effectively disrupt tumor growth and immune suppression, supporting its potential application in melanoma therapy [126]. Additionally, Ocoxin has been identified as a promising agent capable of significantly reducing Gal-1 secretion by melanoma cells, potentially enhancing treatment efficacy [127].

Further supporting the role of Gal-1 in therapy resistance, recent research in prostate cancer has demonstrated that inhibiting the Gal-1 and androgen receptor (AR) axis enhances the efficacy of enzalutamide treatment in enzalutamide-resistant prostate cancer [86]. This finding underscores the broader significance of Gal-1 in tumor resistance mechanisms and suggests that targeting Gal-1 in melanoma could similarly improve responses to existing treatments. Moreover, the activation of Gal-1/NRP1 autocrine signaling enables melanoma cells to sustain oncogenic activity independently of BRAF kinase function, further complicating treatment strategies and emphasizing the need for targeted interventions [119].

Notably, a neutralizing anti-Gal-1 monoclonal antibody developed by Pérez Sáez et al. demonstrated strong angioregulatory and immunomodulatory effects, blocking Gal-1–glycan interactions, restoring T-cell viability, and reducing tumor-induced angiogenesis [128]. These findings highlight the therapeutic potential of Gal-1 blockade in overcoming immune suppression and enhancing anti-tumor responses.

By integrating Gal-1-targeting approaches with advanced imaging and combination therapies, researchers can optimize melanoma treatment strategies, mitigate therapy resistance, and improve patient outcomes across multiple cancer types.

### Adverse Effects of Inhibiting Galectin-1

#### Role in Stress Regulation and Neural Development

While inhibiting Gal-1 presents a valuable opportunity in melanoma therapy, its systemic functions must be carefully considered due to potential adverse effects. Studies on Lgals1−/− and Lgals3−/− mice indicate that the absence of Gal-1 leads to reduced anxiety-like behavior, suggesting its role in stress regulation [129]. Additionally, it plays a vital function in promoting neurite extension, which is essential for axonal growth and neural repair [130]. Gal-1 also promotes neurite extension, essential for axonal growth and regeneration. Expanding on its neuroprotective potential, Gal-1 has been shown to attenuate neuroinflammation and cognitive impairment in various pathological contexts. In HIV-1-infected microglia, both exogenous Gal-1 and a gold nanoparticle-Gal-1 nanoplex reduced proinflammatory signaling by shifting the nitric oxide–arginase balance and downregulating cytokine production, fostering a neuroprotective phenotype [131]. Similarly, Gal-1 administration in a rodent model of status epilepticus reduced neuronal loss, preserved blood-brain barrier integrity, and suppressed glial activation, while enhancing anti-inflammatory cytokine expression [132]. In aged mice, Gal-1 treatment mitigated perioperative neurocognitive disorders by inhibiting microglial activation, reducing IL-1β and TNF-α expression, and restoring synaptic plasticity [133]. Further, Gal-1 was shown to promote vascular remodeling and enhance cerebral blood flow following ischemic stroke, supporting its role in post-injury neural recovery [134]. Complementing these preclinical findings, clinical data suggest a link between Gal-1 and cognitive performance: individuals with bipolar disorder showed altered serum Gal-1 levels that were significantly correlated with cognitive function, indicating the potential involvement of Gal-1 in neuropsychiatric regulation and cognition [135]. Collectively, these findings reinforce the role of Gal-1 as a key regulator of neural homeostasis, stress resilience, vascular adaptation, and cognitive integrity across both physiological and pathological states.

#### Cardioprotective Functions

Beyond the nervous system, Gal-1 exerts cardioprotective effects by mitigating inflammation and apoptosis in myocardial tissues, highlighting its potential role in preventing heart disease [136]. In early acute myocardial infarction, Gal-1 is rapidly upregulated in infarcted myocardial tissue and is associated with inflammatory cell infiltration, suggesting its involvement in modulating the early inflammatory response and limiting tissue damage [137]. Gal-1 has been shown to reduce coronary artery inflammation and circulating pro-inflammatory cytokines, likely contributing to immune regulation in cardiovascular disease [138]. It also directly interacts with the CaV1.2 calcium channel, promoting its degradation and thereby reducing calcium influx and associated pro-inflammatory signaling [139]. More specifically, Gal-1 attenuates cardiomyocyte hypertrophy through splice-variant-specific modulation of CaV1.2, further underscoring its regulatory role in calcium homeostasis and cardiac remodeling[140]. Furthermore, Gal-1 inhibits inflammation and calcification in valvular interstitial cells, with its expression influenced by estrogen, suggesting a hormone-sensitive protective role in aortic stenosis [141]. These findings emphasize the key role of Gal-1 in maintaining cardiovascular health, underscoring the need for caution when considering Gal-1 inhibition in cancer therapy.

#### Modulation of Immune Response

Gal-1 contributes to immune tolerance and tissue protection by modulating inflammatory responses, which is crucial for preventing excessive immune activation [142]. In the immune system, Gal-1 is essential for the regulatory function of B cells, facilitating IL-10 production and suppressing effector T-cell response [66]. Loss of Gal-1 impairs B cell–mediated suppression of inflammation and fails to protect against autoimmune pathology in vivo, highlighting its role in maintaining immune homeostasis. Additionally, Gal-1 levels positively correlate with inflammatory markers and regulatory T cell frequencies in children with type 1 diabetes and/or celiac disease, supporting its role as a biomarker of immune regulation in human autoimmune conditions [143]. Silencing Gal-1 and Gal-3 in both immature and mature dendritic cells enhances T cell activation and interferon-γ production, reinforcing the broader immunosuppressive functions of Gal-1 across multiple antigen-presenting cell types [144]. Given these essential physiological and immunomodulatory roles, therapeutic strategies targeting Gal-1 must be designed carefully to balance the benefits of melanoma treatment with the potential systemic risks associated with its inhibition.

### Translating Research to Human Models

Despite compelling preclinical evidence supporting Gal-1 as a therapeutic target in cancer, no FDA-approved Gal-1–targeting agents are currently available in clinical practice. OTX008, a small-molecule Gal-1 inhibitor, demonstrated anti-proliferative and anti-invasive effects in preclinical studies [145] and entered a Phase I clinical trial. However, the trial revealed dose-limiting toxicities, including neurological adverse events and poor local tolerance at the injection site, leading to its discontinuation [146]. Additionally, broader galectin inhibitors such as GR-MD-02 (Belapectin), which primarily targets Gal-3, have shown potential in modulating the tumor microenvironment and improving immune function in preclinical models [147].

More recently, Gal-3 inhibitors have been evaluated in combination with immune checkpoint inhibitors in melanoma and other cancers. A Phase 1b trial investigated belapectin with pembrolizumab in patients with advanced metastatic melanoma and reported a disease control rate of 56% among melanoma patients, including one partial response and four cases of stable disease. The combination was well tolerated and suggested potential synergy. Similarly, Galecto Inc. initiated a Phase 2 trial in 2022 to investigate GB1211, an oral Gal-3 inhibitor, in combination with pembrolizumab for metastatic melanoma and head and neck squamous cell carcinoma, based on preclinical evidence of enhanced ICI efficacy with Gal-3 blockade.

Despite these advances, specific Gal-1–targeting agents remain underdeveloped, and translational barriers persist due to redundancy among galectin family members, the complex and context-dependent roles of Gal-1 in immune modulation, and challenges in selective tumor targeting. Future research should prioritize the development of more selective, bioavailable Gal-1 inhibitors, the identification of robust biomarkers to stratify responsive patient populations, and the evaluation of rational combination strategies with existing immunotherapies. Rigorous clinical validation and improved delivery platforms will be essential to advancing Gal-1–targeted therapies toward regulatory approval and clinical integration.

### Gaps in Current Research

#### Contributions of Gelectin-1

Although the role of Gal-1 in melanoma progression and immune modulation has been widely recognized, several key limitations in the literature remain. While existing studies demonstrate the capacity of Gal-1 to modulate immune responses and protect tumor cells from cytotoxic insults, the precise molecular mechanisms behind these effects are still unclear. Specifically, the distinction between the intracellular versus extracellular functions of Gal-1 requires deeper exploration, as this could have major implications for targeted therapeutic development [148]. Moreover, while Gal-1 is the most studied member of the galectin family in melanoma, the roles of other galectins, such as Gal-3 and Gal-9, remain underexplored and may contribute to redundancy or compensatory mechanisms in tumor progression and immune evasion [119].

The contribution of Gal-1 to chemotherapy resistance in melanoma also presents a significant research gap. While some reports suggest that Gal-1 promotes resistance to programmed cell death and therapeutic agents, the pathways driving intrinsic versus acquired resistance have not been sufficiently delineated [119]. Clarifying these distinctions will be essential for developing therapies that not only block Gal-1 but also prevent the emergence of resistant melanoma subclones. Additionally, the immunosuppressive function of Gal-1 has primarily focused on T-cell interactions; further studies are needed to assess its impact on other immune populations such as dendritic cells, macrophages, and myeloid-derived suppressor cells [149]. Addressing these gaps is crucial to fully leveraging Gal-1 as a therapeutic target.

#### Tumor Microenvironment

The TME plays a pivotal role in melanoma progression and therapeutic resistance. Recent studies have highlighted several critical areas requiring further exploration, particularly regarding the role of Gal-1. While Gal-1 has been shown to contribute to immune suppression within the TME, especially by inhibiting T-cell activity, its broader impact on other immune cells, such as macrophages and dendritic cells, remains largely unexplored [149]. The interactions between Gal-1 and non-T-cell populations could present new therapeutic targets, but studies addressing this gap are still limited. Additionally, the heterogeneous nature of the TME complicates the study of the effects of Gal-1, as its role may vary depending on the tissue context and the specific molecular signals present within different melanoma subtypes.

Moreover, the role of TME in drug resistance further complicates treatment strategies. Melanoma cells exploit various mechanisms within the TME to evade immune surveillance, such as recruiting immunosuppressive cells and secreting immunomodulatory factors, which significantly hinder the effectiveness of immunotherapy [150]. Additionally, the TME can modulate drug metabolism and efficacy, leading to resistance against both targeted therapies and chemotherapy [150]. Addressing these factors will be essential for overcoming treatment resistance. As melanoma therapies evolve, understanding the complexities of the TME and developing strategies to modulate its influence will be key to improving clinical outcomes in melanoma treatment.

#### Metastatic Melanoma

Patients with advanced melanoma, particularly those with brain metastases, face poor prognoses due to therapeutic resistance and the distinct immune microenvironments present at metastatic sites. Emerging evidence suggests that the TME in metastatic melanoma differs significantly from that of primary tumors—especially in the brain, where a highly immunosuppressive milieu impairs the efficacy of systemic therapies [151]. Studies on melanoma brain metastases have identified unique immune features, including altered T-cell distribution and phenotype, further emphasizing the complexity of the intracranial TME. However, the role of Gal-1 in shaping this immunosuppressive landscape, particularly within the brain TME, remains poorly understood. Given the established function of Gal-1 in immune regulation and its involvement in promoting immune exclusion, further research is needed to elucidate how Gal-1 may contribute to immune evasion and therapeutic resistance in melanoma brain metastases. This complex immune environment presents a major barrier to effective treatment, highlighting the importance of investigating Gal-1 as a potential therapeutic target in the context of brain metastases.

#### Therapeutic Development

While Gal-1 has shown promise as a therapeutic target, more studies are needed to validate and optimize Gal-1 inhibitors in melanoma. Combination therapies that integrate Gal-1 blockade with existing treatments such as immune checkpoint inhibitors (ICIs) have shown synergistic effects in preclinical models, enhancing T-cell infiltration and overcoming resistance [149]. Future research should explore the optimal timing, dosing, and sequencing of such combinations in both early-stage and metastatic melanoma.

In addition, the development of non-invasive imaging tools like Gal-1–targeted PET imaging holds promise for predicting therapeutic response and guiding personalized treatment strategies. A recent study demonstrated that Gal-1 PET imaging could successfully monitor Gal-1 expression in melanoma, offering a potential companion diagnostic to assess patient eligibility for Gal-1–targeted therapies [125]. These tools could be instrumental in identifying patients most likely to benefit from combination treatments and tailoring therapies to individual tumor profiles.

#### Clinical Trials

Despite preclinical progress, Gal-1-targeted therapies have yet to be robustly tested in human clinical trials. Most current data are limited to in vitro models or animal studies, with a lack of multi-center, phase II/III trials to assess safety and efficacy in diverse patient populations. There is a clear need for clinical investigations that explore Gal-1 inhibition alone and in combination with ICIs, MAPK inhibitors, or chemotherapy agents. These studies should account for patient heterogeneity, including variations in Gal-1 expression, tumor stage, and immune status, to evaluate the generalizability and real-world application of Gal-1–based interventions.

### Conclusions

Gal-1 plays a role in glycan-dependent immune suppression, angiogenesis, and therapeutic resistance, identifying it as a potential key therapeutic agent in melanoma. The role of Gal-1 in immune modulation, through interaction with T cell, causing T-cell exhaustion and recruitment of myeloid-derived suppressor cells and tumor-associated macrophages, helps create an immunosuppressive tumor microenvironment. Additionally, Gal-1 promotes angiogenesis despite anti-VGEF signaling, allowing tumors to resist anti-VGEF therapy. Further, Gal-1 resists therapies through PI3K/Akt/mTOR activation, CoREST-mediated phenotype switching, CCR2^+^ macrophage cues, and AURKA-driven instability. The ability of Gal-1 to promote tumor microenvironments and resist therapies makes it a powerful therapeutic target for melanoma. Recent research has considered addressing each of these strategies, including a Gal-1 blockage with anti-PD-1 and targeting the VEGF-pathway and P13K/Akt nodes. Additionally, researchers have considered utilizing Gal-1 PET and tissue glycosylation to monitor patients, and evaluating tumors with BRAF/MEK inhibitors to prevent Gal-1 mediated escape. However, consideration of the cardio- and neuro-protective effects of Gal-1, role in wound healing, and immune system functions is necessary for further therapeutic development by creating strategies to target tumors. Targeting Gal-1 within melanoma, through biomarker-driven patient selections, offers a strong therapeutic pathway to mitigate immune exclusion and resistance to other therapies, warrants further research and translational efforts.

## Figures and Tables

**Table 1:** Comprehensive summary of Galectin subtypes organized by their structural class, outlining their tissue distribution within humans and cellular functions. This table details the key physiological and pathological roles of each Galectin. Notably, many Galectins (Gal-1, Gal-3, and Gal-9) have major functions in immune tolerance, fibrosis, and cancer progression.

Class	Galectin Type	Tissue Distribution	Cellular Effects
**Prototypical**	Galectin-1	Various tissues throughout human body	Immune system regulation, angiogenic regulation, wound healing, fibroblast proliferation, etc.
	Galectin-2	Primarily within the gastrointestinal tract, placenta, and cardiovascular system [13]	Apoptosis, inflammation [14, 15], immune system regulation [16]
	Galectin-5	Not found in humans [17]	
	Galectin-7	Stratified epithelia, skin, fetal heart, gastrointestinal tract, breast myoepithelial cells [18], cornea [19]	Apoptosis [3], cell migration, proliferation [18], wound re-epithelization [19], immune system regulation [20]
	Galectin-10	Primarily found in eosinophils, but is also expressed in other immune cells [21]	Produces Charcot-Leyden crystals [22], immune system regulation [23]
	Galectin-11	Not found in humans	
	Galectin-13	Placenta, as well as in the spleen, bladder, and kidney [24] to a lesser extent	Vascular remodeling, apoptosis, immune modulation [25]
	Galectin-14	Placenta	Immune regulation
	Galectin-15	Not found in humans	
	Galectin-16	Placenta	Immune regulation
**Tandem-repeat**	Galectin-4	Gastrointestinal tract, hippocampal and cortical neurons	Cell adhesion [19], host-gut microbe interactions, prevents pathogen spreading [26], tumor suppression [27], cell cycle regulation, T cell apoptosis [28], protein trafficking [29]
	Galectin-6	Not found in humans	
	Galectin-8	Gastrointestinal tract [30], placenta [31], central nervous system [32], liver [30], kidney [33], cardiovascular system [34], lung [35], spleen, thymus [34]	Immune regulation, inflammatory responses, autophagy [36], apoptosis [34], thrombosis, angiogenesis [37], regulate cell adhesion and growth [35]
	Galectin-9	Skin [38], gastrointestinal system [39], liver [40], immune organs [41], lung [42], kidney [43], brain [44]	Immunomodulation [42], attracts eosinophils [45], T helper cell apoptosis [46], neutrophil adhesion [47], cellular proliferation, epithelial repair [48], inflammation [49]
	Galectin-12	Primarily, found in adipose tissue [50], but it is also found in immune cells, skin [51], liver [52]	Inflammation [50], apoptosis, cell cycle regulation, modulates glucose and insulin sensitivity, immune system regulation [53]
**Chimera**	Galectin-3	Various immune cells [54], skin [55], gastrointestinal tract, lung, nervous system [56], kidney, thymus, lymph nodes [57], liver [58], epiphyseal cartilage [59], bone [60], cardiovascular system [61], thyroid [62], endometrium [63], testes [64], ovary [64]	Immunomodulation, embryogenesis, angiogenesis, cell migration, apoptosis, wound repair, host-pathogen interactions [54]
